# Nuclear quantum effect and H/D isotope effect on Cl· + (H_2_O)_*n*_ → HCl + OH·(H_2_O)_*n*−1_ (*n* = 1–3) reactions†

**DOI:** 10.1039/c8ra02679c

**Published:** 2018-05-10

**Authors:** Keita Sugiura, Masanori Tachikawa, Taro Udagawa

**Affiliations:** Department of Chemistry and Biomolecular Science, Faculty of Engineering, Gifu University Yanagido 1-1 Gifu 501-1193 Japan udagawa@gifu-u.ac.jp +81-58-293-2575; Quantum Chemistry Division, Graduate School of NanoBioScience, Yokohama City University Seto 22-2, Kanazawa-ku Yokohama 236-0027 Japan

## Abstract

Cl· + (H_2_O)_*n*_ → HCl + OH·(H_2_O)_*n*−1_ (*n* = 1–3) reactions are fundamental and important ones in atmospheric chemistry. In this study, we focused on the nuclear quantum effect (NQE) of the hydrogen nucleus on these reactions with the aid of the multicomponent quantum mechanics (MC_QM) method, which can directly take account of NQE of light nuclei. Our study reveals that the NQE of the hydrogen nucleus lowers the activation barriers of the reactions and enhances the catalytic effects of second and third water molecules. In particular, we find that (i) the NQE of the proton removes the activation barrier of the reverse reaction of HCl + OH· → Cl· + H_2_O, and (ii) the catalytic effect of the third water molecule appears in only our MC_QM calculation. We also analyze the H/D isotope effects on these reactions by using the MC_QM method.

## Abstract 2

Cl·+ (H_2_O)*_n_* → HCl + OH(H_2_O)*_n_*_−1_ (*n* = 1–3) reactions have been investigated using multicomponent quantum mechanics method, which can take account of the nuclear quantum effect of proton and deuteron.

## Graphical abstract

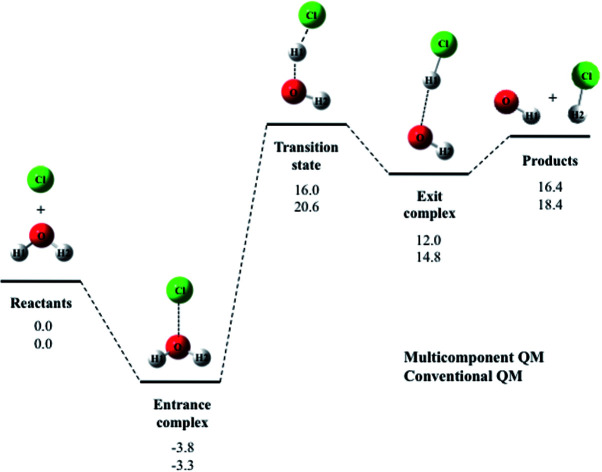

## 1. Introduction

Water is indispensable to living organisms and also has important roles in various chemical reactions as a solvent. For instance, it is known that solvent water molecules lower the activation energy of proton transfer reactions by acting as a proton shuttle in the relay mechanism.^1^ Thus, reactions involving water molecules have attracted a lot of attention in a wide variety of branches of chemistry, such as atmospheric chemistry, catalytic chemistry, surface chemistry, biochemistry, and so on.^2–4^ In particular, reactions with halogen atoms have attracted special interest. For example, the reactions with Cl·, which is known as the major cause of the serious environmental problem of “ozone holes” in Antarctica,^5^ have been earnestly studied by several groups.^5–7^ Recently, Li and coworkers reported the potential energy profiles for Cl· + (H_2_O)_*n*_ (*n* = 1 and 2) reactions using the “gold-standard” CCSD(T)/cc-pVQZ method. As Li and coworkers mentioned,^8^ investigations of the reactions with a few water molecules are important as an initial step to understand chemical reactions in water at the molecular level. Therefore, it is important to analyze such small basic chemical reactions with a high-accuracy method, such as the CCSD(T)/cc-pVQZ method. They reported that the second water molecule acted as a catalyst, that is, the second water molecule lowered the activation energy for the hydrogen transfer reaction in the Cl· + H_2_O reaction.

On the other hand, the importance of the nuclear quantum effect (NQE) of a light nucleus, such as a proton, has been recognized in various fields in tandem with the recent advances of experimental techniques and computational methods. The NQE of a proton provides significant effects in several reactions and systems, in which the hydrogen atom takes a central role, such as hydrogen transfer reactions, hydrogen absorption reactions, hydrogen-bonded systems, and so on. In addition, NQE is one of the main contribution factors to form low-barrier hydrogen-bonded (LBHB) systems,^9–14^ which have been considered to be important for functional expression of some proteins. Also, it is known that deuterium substitution often induces significant hydrogen/deuterium (H/D) isotope effect, since a deuterium is a twice-heavier isotope of hydrogen. For example, the phase transition temperature of hydrogen-bonded ferroelectric material drastically (>100 K) changes by replacing a hydrogen-bonded hydrogen atom with deuterium,^15^ and the reaction rate constant of 1,5-sigmatropic hydrogen transfer reaction in 1,3-pentadiene is more than 10 times faster than the transfer in deuterated system.^2^ In addition, deuterium substitution of solvent molecules (solvent H/D isotope effect) is also important for molecular properties. For example, the critical temperature of lens protein of γB-crystallin in D_2_O is 16 K higher than that in H_2_O.^16^ Since Cl· + (H_2_O)_*n*_ reactions are important as an initial step to understand chemical reactions in solution, as mentioned above, we believe that the analyses of the Cl· + (D_2_O)_*n*_ reactions are also important toward understanding the chemical reactions involving heavy water.

However, the conventional quantum mechanical (QM) calculations usually can not take account of nuclear motion based on the Born–Oppenherimer (BO) approximation.^17^ In the framework of BO approximation, only electronic Schrödinger equation is solved under the field of clamped nuclear charges. Therefore, it is difficult for the conventional QM methods to directly reflect NQE on electronic structure. On the other hand, we have recently proposed multicomponent QM (MC_QM) methods,^18–21^ which can directly reflect NQE of light nuclei on electronic structures. We have already successfully analyzed H/D isotope effect on geometries (H/D geometrical isotope effect: GIE) in various hydrogen-bonded systems and H/D kinetic isotope effect (KIE) in several hydrogen transfer reactions using our MC_QM methods.^22,23^ In addition, Ishimoto and Koyama successfully revealed the dynamic behavior of nuclear wavefunction of hydrogen nucleus in H_5_O_2_^+^ and its isotopomers by performing molecular dynamics simulation on the MC_QM effective potential energy hypersurface.^24^ Quite recently, we have proposed MC_QM-climbing image-nudged elastic band (CI-NEB) method^18^ by combining our MC_QM method with CI-NEB method,^25^ and have successfully analyzed H/D isotope effect in proton/hydrogen transfer reactions^18,22,23^ and H/D isotope effect in isomerization reactions^23^ including NQE of light nuclei with the aid of MC_QM-CI-NEB method.

Although CCSD(T) calculation can predict electronic energy and molecular geometries quite accurately, NQEs of light nuclei are not taken into account within the framework of BO approximation. For the systems and the reactions, in which NQEs of light nuclei are remarkable, we have to not only improve the accuracy of electronic structure calculations but also adequately include NQE of light nuclei beyond the BO approximation. Thus, the main subject of the present study is to reveal NQE of hydrogen nuclei on Cl· + (H_2_O)_*n*_ (*n* = 1–3) reactions using MC_QM-CI-NEB method. First, we investigate the performance of several major exchange–correlation density functionals to obtain the reliable optimized geometries of the stationary points structures in Cl· + (H_2_O)_*n*_ (*n* = 1–3) reactions. Because one of the efficient ways to obtain reliable energies is calculating CCSD(T) energies at the geometries optimized by DFT method with the appropriate exchange–correlation functional. Although it is known that DFT calculations can provide reasonable energy and molecular geometries, the accuracy of DFT calculations strongly depends on the selection of exchange–correlation functional. We, thus, try to determine the best exchange–correlation functional for Cl· + (H_2_O)_*n*_ (*n* = 1–3) reactions by calculating CCSD(T) energy at the geometry optimized by DFT (denoted as CCSD(T)//DFT), and compare it with the energy obtained by pure CCSD(T) calculation. Then the NQE on Cl· + (H_2_O)_*n*_ and Cl· + (D_2_O)_*n*_ (*n* = 1–3) reactions have been analyzed using MC_CCSD(T) and MC_DFT calculations.

In the next section, we briefly introduce our MC_QM method and CI-NEB method. Computational details to analyze Cl· + (H_2_O)_*n*_ (*n* = 1–3) reactions are given in Sec. 3. The computational details for MC_QM and MC_QM-CI-NEB calculations are also given in Sec. 3. Performance of the several density functionals to reproduce CCSD(T) stationary point geometries is discussed in Sec. 4-1. The NQE and H/D isotope effect on the reactions are discussed in Sec. 4-2. Finally, some concluding remarks are given in Sec. 5.

## 2. Theory

### 2.1. Multicomponent quantum mechanics (MC_QM) method

In this section, we would like to introduce our MC_QM method briefly (see ref. 19–21 and references therein for more detailed information). In our MC_QM approach, the total Hamiltonian for system containing N_e_-electrons, N_p_-quantum nuclei, and M-classical nuclei, is expressed as1
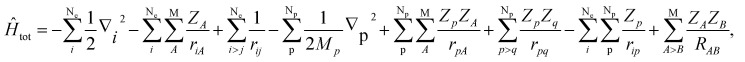
where the indices of *i* and *j* refer to the electrons, *p* and *q* to the quantum nuclei, and *A* and *B* to the classical nuclei. In addition, *Z*_*A*_ represents nuclear charge of *A*th nucleus, and *M*_*p*_ is the mass of *p*th quantum nucleus. The first to third terms are the conventional terms for electrons, and the fourth to sixth terms are those for quantum nuclei. The seventh term represents the coulombic interaction between electron and quantum nucleus, and the last term represents the classical nuclear repulsion.

The total wavefunction *Ψ*_0_ is represented by a simple product of electronic and nuclear wavefunction in the Hartree–Fock level of MC_QM method,

2 *Ψ*_0_ = *Φ*^e^_0_*Φ*^p^_0_,

where *Φ*^e^_0_ and *Φ*^p^_0_ are the electronic and the nuclear wavefunctions, respectively. The effective one-particle (one-electron and one-nucleus) operators are3
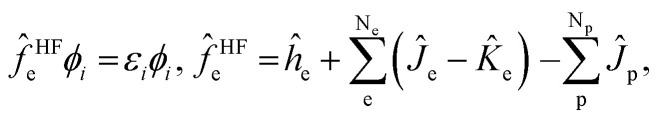
4
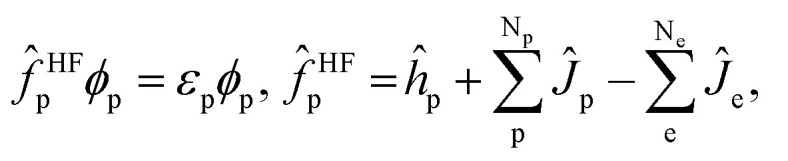
where *ĥ*_e_ and *ĥ*_p_ are one-particle operators for electron and quantum nucleus, *Ĵ*_e_ and 
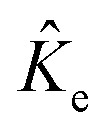
 are the electronic Coulomb and exchange operators, and *Ĵ*_p_ is the Coulomb operator for quantum nuclei. The nuclear exchange term is ignored in this study due to its small contribution.^26^ To solve the electronic Fock equation, we use linear combination of Gaussian-type functions (LCGTFs) for expanding electronic molecular orbitals (MOs),5
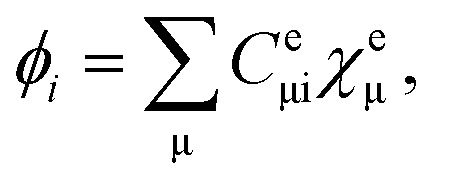
where *C*^e^_μi_ is the LCGTF coefficients for an electron, and *χ* is the Gaussian-type function (GTF). Although quantum nuclear MOs should be expanded using a suitable number of GTFs as well as the electronic MOs, we have already demonstrated that nuclear quantum effect is adequately taken into account by using only single s-type GTF as nuclear basis function.^18,20,22,23^

It should be noted here that new types of many-body effects, such as electron–quantum nucleus and quantum nucleus–quantum nucleus correlation effects arise in MC_QM scheme. To improve accuracy, we have to evaluate these correlation effects as well as the conventional electron–electron one. However, it is worth mentioning that the contributions from electron–quantum nucleus and quantum nucleus–quantum nucleus correlations are much smaller than that from electron–electron one for typical molecular systems, and we have shown that H/D isotope effects in various hydrogen-bonded systems and hydrogen transfer reactions can be analyzed evaluating only electron–electron correlation effect in MC_QM calculations.^18–23^ In particular, we have already demonstrated that H/D isotope effects in similar reactions of F· + (H_2_O)_*n*_ → HF + OH·(H_2_O)_*n*−1_ (*n* = 1–3) can be adequately analyzed even if only electron–electron correlation effect is evaluated using established density functionals for electrons and CCSD(T) method.^22^ Therefore, we also evaluate only electron–electron correlation effect in the present MC_QM calculations.

### 2.2. Climbing image-nudged elastic band (CI-NEB) method

To find out the transition state (first order saddle point) structure on MC_QM effective potential energy surface (PES), we have carried out MC_QM-CI-NEB calculations. Here, we would like to briefly introduce the algorithm of the CI-NEB method.^25,27,28^

In the NEB calculations, the total force acting on an image is calculated as the sum of the spring force along the tangent and the true force perpendicular to the tangent

6 **F**_*i*_ = **F**^s^_*i*_|_||_ − ∇*V*(**R**_*i*_)|_⊥_.

The first term of the right-hand side of eqn (6) corresponds to the spring force, which is given as

7 **F**^s^_*i*_|_||_ = *k*(|**R**_*i*+1_ − **R**_*i*_| − |**R**_*i*_ − **R**_*i*−1_|)τ̂_*i*_,

where *k* is the spring constant. We adopted the variable spring constants^27^ to efficiently improve the resolution of the vicinity of the transition state8
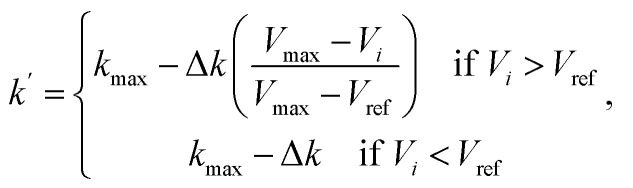
where Δ*k* = *k*_max_ − *k*_min_, *V*_max_ is the highest energy for the whole elastic band, *V*_*i*_ is the higher energy of the two images connected by spring *i*. According to Henkelman's original paper,^27^ we chose the energy of the higher energy endpoint of the MEP as *V*_ref_.

The second term of the right-hand side of eqn (6) is given as

9 ∇*V*(**R**_*i*_)|_⊥_ = ∇*V*(**R**_*i*_) − ∇*V*(**R**_*i*_)τ̂_*i*_τ̂_*i*_.

τ̂ in eqn (7) and (9) is the unit vector along the reaction path. We used Henkelman's revised tangent^28^ in this study. The revised tangents are defined as10
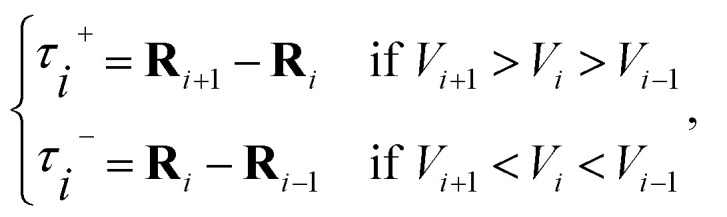
where **R**_*i*_ and *V*_*i*_ are the position vector and energy of *i*-th image, respectively. For the image at an energy minimum (*V*_*i*+1_ > *V*_*i*_ < *V*_*i*−1_) or at an energy maximum (*V*_*i*+1_ < *V*_*i*_ > *V*_*i*−1_), the tangent becomes11
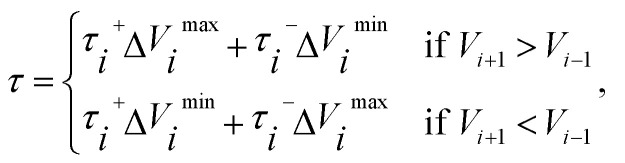
where12
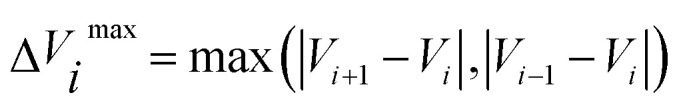
and13
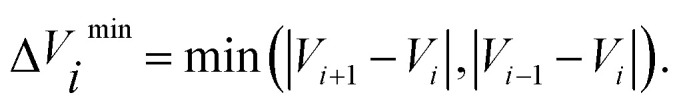


For the image with the highest energy, the total force is evaluated according to the following expression instead of eqn (6)

14 **F**_*i*_max__ = ∇*V*(**R**_*i*_max__) − 2∇*V*(**R**_*i*_max__)τ̂_*i*_max__τ̂_*i*_max__.

It should be noted here that we can obtain both minimum energy path and transition state structure on the MC_QM effective PES by minimizing (CI-)NEB forces in eqn (6) and (14) using MC_QM method. We have already demonstrated that MC_QM-CI-NEB approach adequately provides effective transition state structure on the MC_QM PES for several reactions.^18,22,23^

## 3. Computational detail

To investigate the performance of density functionals for geometry optimization, all stationary points of Cl· + (H_2_O)_*n*_ → HCl + OH(H_2_O)_*n*−1_ (*n* = 1–3) reactions were optimized using five major density functionals (B3LYP, CAM-B3LYP, M06, ωB97XD, and MPW1K) with correlation-consistent cc-pVTZ electronic basis set. Then, we calculated CCSD(T)/cc-pVQZ energy at the geometry optimized by DFT (CCSD(T)/cc-pVQZ//DFT/cc-pVTZ). It is known that diffuse functions are often important to describe hydrogen-bonded systems. Thus, first, we investigated the importance of diffuse functions for calculating relative potential energy profile for Cl· + H_2_O reaction. The calculated relative energies and the optimized geometrical parameters obtained with cc-pVXZ and aug-cc-pVXZ (X = T or Q) electronic basis sets are shown in Fig. S1 in ESI.† The activation energy obtained by CCSD(T)/aug-cc-pVQZ//ωB97XD/aug-cc-pVTZ calculations (20.7 kcal mol^−1^) is almost equal to that by CCSD(T)/cc-pVQZ//ωB97XD/cc-pVTZ calculations (20.6 kcal mol^−1^). Therefore, the effect of diffuse functions is also negligible in Cl· + H_2_O reactions, as in the case of F + H_2_O ones.^22^

Next, to analyze HCl + OH·(H_2_O)_*n*−1_ → Cl· + (H_2_O)_*n*_ and DCl + OD·(D_2_O)_*n*−1_ → Cl· + (D_2_O)_*n*_ (*n* = 1–3) reactions including NQE of proton and deuteron, we performed MC_CCSD(T)/cc-pVQZ//MC_DFT/cc-pVTZ calculations. The effective transition state structure on MC_QM effective potential energy hypersurface is obtained by MC_DFT-climbing image-nudged elastic band (CI-NEB)^18^ calculations. In MC_QM calculations, all protons and deuterons were treated as quantum wavefunction. We adopted single s-type Gaussian-type functions (GTFs), exp{−*α*(*r* − *R*)^2^}, as nuclear wavefunction. The *α* value in GTF is the orbital exponent value and determines the spatial distribution of nuclear wavefunction. We used average *α* values (*a*^H^_ave_ = 24.1825 and *a*^D^_ave_ = 35.6214) for geometry optimizations.^29–31^ To refine the spatial distribution of nuclear wavefunctions and the total energy, we optimized *α* (*α*_opt_) values by MC_ωB97XD/cc-pVQZ//MC_ωB97XD/cc-pVTZ calculations. Then we calculated the more reliable MC_CCSD(T)/cc-pVQZ//MC_ωB97XD/cc-pVTZ energies with these *α*_opt_ values. We have confirmed that *α*_opt_ values obtained by MC_ωB97XD/cc-pVQZ and MC_CCSD(T)/cc-pVQZ calculations are similar to each other and MC_CCSD(T)/cc-pVQZ energies obtained using MC_ωB97XD/cc-pVQZ *α*_opt_ values and using MC_CCSD(T)/cc-pVQZ *α*_opt_ values are also quite similar to each other (see, Fig. S2 in ESI†). All calculations were performed with the modified version of GAUSSIAN 09 program package.^32^

## 4. Results and discussion

### 4.1. DFT calculations for Cl· + (H_2_O)_*n*_ → HCl + OH·(H_2_O)_*n*−1_ (*n* = 1–3) reactions

To determine the best density functional for the reactions of Cl· + (H_2_O)_*n*_ → HCl + OH·(H_2_O)_*n*−1_ (*n* = 1–3), we optimized the stationary points structures of Cl· + H_2_O → HCl + OH· reaction using five major density functionals (B3LYP, CAM-B3LYP, M06, ωB97XD, and MPW1K) and compared the optimized interatomic distances in DFT-optimized geometries with those in CCSD(T)/cc-pVQZ-optimized geometry.

Table 1 lists the optimized geometrical parameters in the stationary points structures of Cl· + H_2_O → HCl + OH· reaction obtained by DFT/cc-pVTZ calculations. Guo's CCSD(T)/cc-pVQZ values^6^ are also listed in Table 1 for comparison. We can find that all five density functionals can reproduce the CCSD(T)/cc-pVQZ interatomic distance well, except for transition state. The O⋯H1 distances in transition state are 1.515 Å and 1.413 Å in B3LYP and M06 geometries, respectively. Both of them were clearly longer than that in CCSD(T) geometry (1.324 Å). Also, Cl⋯H1 distance of 1.345 Å in B3LYP geometry is slightly shorter than that in CCSD(T) one (1.391 Å).

**Table 1:** Optimized geometrical parameters (Å) of the stationary point structures of Cl· + H_2_O → HCl + OH· reaction obtained by DFT/cc-pVTZ methods

Method	Reactant	Entrance complex			Transition state			Exit complex			Products	
	R(O–H1)	R(O–H1)	R(O⋯Cl)	R(Cl⋯H1)	R(O–H2)	R(O⋯H1)	R(Cl⋯H1)	R(O–H2)	R(O⋯H1)	R(Cl⋯H1)	R(O–H2)	R(Cl–H1)
B3LYP	0.961	0.965	2.508	2.653	0.975	1.515	1.325	0.976	1.979	1.297	0.974	1.283
CAM-B3LYP	0.960	0.963	2.482	2.681	0.973	1.396	1.368	0.975	1.959	1.293	0.973	1.280
M06	0.957	0.962	2.524	2.678	0.971	1.413	1.371	0.972	1.988	1.297	0.970	1.283
ωB97XD	0.957	0.960	2.501	2.698	0.970	1.386	1.374	0.971	1.977	1.294	0.969	1.280
MPW1K	0.950	0.953	2.498	2.708	0.963	1.303	1.393	0.964	1.979	1.285	0.962	1.273
CCSD(T)/cc-pVQZ^a^	0.958	0.960	2.601	2.845	0.971	1.324	1.391	0.971	2.045	1.285	0.970	1.277

a Ref. 6.

**Fig. 1:**
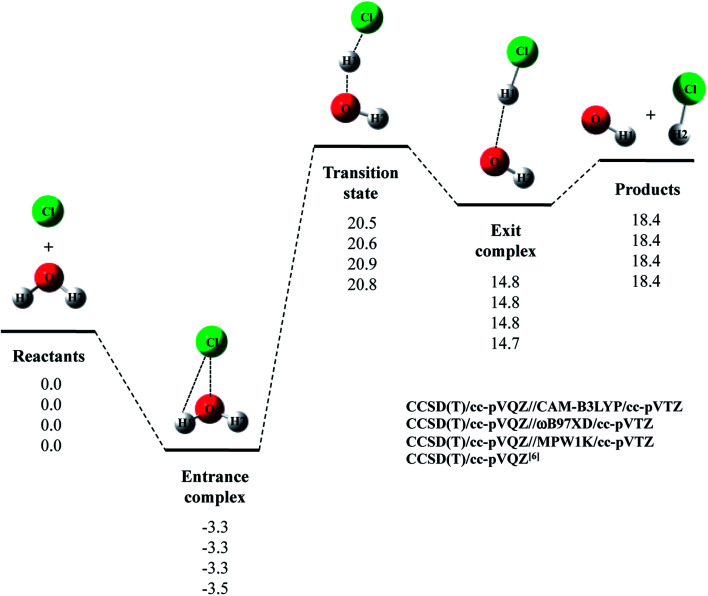
The potential energy profile (kcal mol^−1^) and stationary point structures for Cl· + H_2_O → HCl + OH· reaction obtained by CCSD(T)/cc-pVQZ//DFT/cc-pVTZ calculations. Relative energies are calculated as the energy difference between the energy of each stationary point structure and sum of energies of Cl radical and H_2_O (Reactants).

Next, we evaluated CCSD(T)/cc-pVQZ energy at the geometry optimized with DFT/cc-pVTZ (CCSD(T)/cc-pVQZ//DFT/cc-pVTZ) to obtain high-accuracy energy. The relative energy diagram for the reaction is depicted in Fig. 1. We calculated the CCSD(T)/cc-pVQZ energy at only the CAM-B3LYP, ωB97XD, and MPW1K/cc-pVTZ optimized geometries, since B3LYP and M06 calculations cannot reproduce transition state structure well, as mentioned above. As shown in Fig. 1, all of the CCSD(T)/cc-pVQZ//DFT/cc-pVTZ calculations reproduced CCSD(T)/cc-pVQZ potential energy diagram for Cl· + H_2_O → HCl + OH· reaction well. Difference between the pure CCSD(T) and the CCSD(T)//DFT relative energies are less than 0.3 kcal mol^−1^ for all stationary point structures.

**Fig. 2:**
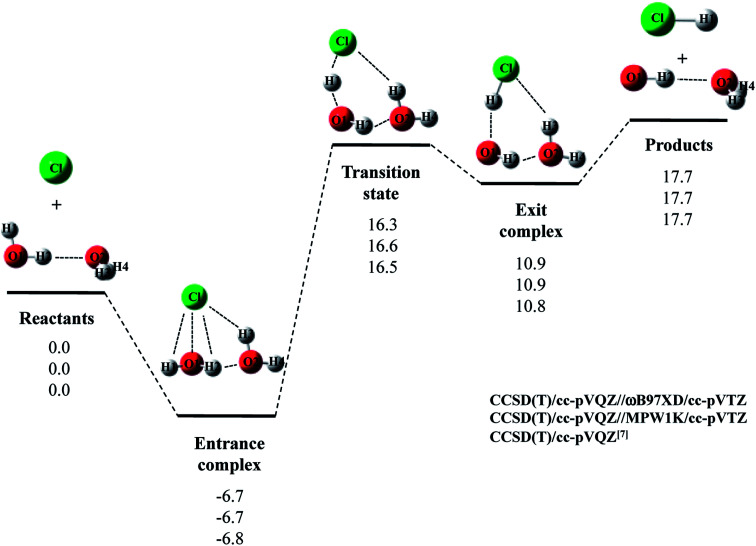
The potential energy profile (kcal mol^−1^) and stationary point structures for Cl· + (H_2_O)_2_ → HCl + OH·(H_2_O) reaction obtained by CCSD(T)/cc-pVQZ//DFT/cc-pVTZ and CCSD(T)/cc-pVQZ^7^ calculations. Relative energies are calculated as the energy difference between the energy of each stationary point structure and sum of energies of Cl radical and (H_2_O)_2_ (reactants).

Next, we would like to focus on Cl· + (H_2_O)_2_ reaction. Fig. 2 shows the optimized structures and Table 2 and 3 list the optimized geometrical parameters of the entrance complex and transition state for the reaction, respectively. The optimized geometrical parameters of other stationary point structures are shown in Tables S1–S3 in ESI.† For Cl· + (H_2_O)_2_ → HCl + OH·(H_2_O) reaction, intermolecular O2⋯H2 distance of entrance complex with B3LYP and CAM-B3LYP calculation is 0.066 Å and 0.081 Å shorter than that with CCSD(T)/cc-pVQZ, respectively. In addition, intermolecular Cl⋯H3 distances with B3LYP, CAM-B3LYP, and M06 calculations are 0.057–0.100 Å shorter than that with CCSD(T)/cc-pVQZ. Thus, these functionals are found to overestimate the interaction energy between Cl(H_2_O) and the second water molecule. On the other hand, the covalent O1–H2 bond lengths in transition state with B3LYP and M06 calculations are 0.016 Å and 0.014 Å longer than that with CCSD(T)/cc-pVQZ, respectively. Meanwhile, the O1–H2 bond length in MPW1K transition state geometry is 0.987 Å, which is almost the same with CCSD(T)/cc-pVTZ value (0.988 Å). However, this is only the accidental coincidence, since MPW1K 0.008 Å underestimate the covalent O–H bond length in water monomer molecule than CCSD(T) (see, reactant in Table 1). Also, B3LYP, M06, and MPW1K calculations gave slightly (about 0.06 Å) shorter intermolecular O2⋯H2 distance compared to CCSD(T). Therefore, we conclude that ωB97XD functional is the best choice to reproduce the CCSD(T)/cc-pVQZ stationary point structures for Cl· + (H_2_O)_2_ → HCl + OH·(H_2_O) reaction.

**Table 2:** Optimized geometrical parameter (Å) of entrance complex for Cl· + (H_2_O)_2_ → HCl + OH·(H_2_O) reaction obtained by DFT/cc-pVTZ methods

Method	R(O1–H1)	R(O1–H2)	R(O2–H3)	R(O2–H4)	R(O1⋯Cl)	R(O2⋯H2)	R(Cl⋯H1)	R(Cl⋯H2)	R(Cl⋯H3)
B3LYP	0.965	0.987	0.968	0.962	2.453	1.765	2.563	2.607	2.654
CAM-B3LYP	0.963	0.983	0.966	0.961	2.410	1.750	2.655	2.590	2.554
M06	0.962	0.980	0.965	0.959	2.476	1.806	2.676	2.622	2.511
ωB97XD	0.960	0.979	0.963	0.958	2.431	1.784	2.659	2.599	2.613
MPW1K	0.953	0.972	0.956	0.951	2.399	1.769	2.650	2.584	2.614
CCSD(T)/cc-pVQZ^a^	0.961	0.974	0.963	0.959	2.452	1.831	2.725	2.637	2.611

a Ref. 7.

**Table 3:** Optimized geometrical parameter (Å) of transition state for Cl· + (H_2_O)_2_ → HCl + OH·(H_2_O) reaction obtained by DFT/cc-pVTZ methods

Method	R(O1–H2)	R(O2–H3)	R(O2–H4)	R(O1⋯H1)	R(O2⋯H2)	R(Cl⋯H1)	R(Cl⋯H3)
B3LYP	1.004	0.969	0.962	1.268	1.710	1.490	2.599
CAM-B3LYP	0.996	0.966	0.961	1.344	1.731	1.406	2.677
M06	1.002	0.969	0.958	1.240	1.715	1.507	2.452
ωB97XD	0.992	0.962	0.958	1.343	1.764	1.407	2.740
MPW1K	0.987	0.956	0.951	1.262	1.717	1.435	2.739
CCSD(T)/cc-pVQZ^a^	0.988	0.963	0.959	1.293	1.777	1.420	2.684

a Ref. 7.

The calculated potential energy diagram for Cl· + (H_2_O)_2_ → HCl + OH·(H_2_O) reaction is shown in Fig. 2. Based on the above results, we calculated CCSD(T)/cc-pVQZ energies at the ωB97XD/cc-pVTZ-optimized geometries. Meanwhile, Li and coworkers claimed that MPW1K is the best choice to analyze a similar reaction of F· + H_2_O → HF + OH·.^33^ Thus, we also calculated CCSD(T)/cc-pVQZ energies at the geometries optimized by MPW1K for comparison. Both of CCSD(T)/cc-pVQZ//ωB97XD/cc-pVTZ and CCSD(T)/cc-pVQZ//MPW1K calculations reproduced CCSD(T)/cc-pVQZ potential energy diagram^7^ well. It should be noted here that in F· + (H_2_O)_2_ → HCl + OH·(H_2_O) reaction, although the CCSD(T)/cc-pVQZ//ωB97XD/cc-pVTZ calculation reproduced the CCSD(T)/cc-pVQZ potential energy diagram well, the CCSD(T)/cc-pVQZ//MPW1K/cc-pVTZ calculation failed to reproduce CCSD(T)/cc-pVQZ potential energy diagram even qualitatively.^22^ Therefore, the performance of exchange–correlation density functional is different even for similar reactions of X· + (H_2_O)_*n*_ (X = F and Cl).

The relative energy of transition state lies about 16 kcal mol^−1^ above the sum of energies of separate reactants, and is about 4 kcal mol^−1^ lower than that of the reaction with water monomer (Fig. 1). Thus, the second water molecule lowers the barrier of Cl· + H_2_O → HCl + OH· reaction, and acts as a catalyst as is the case of F· + (H_2_O)_2_ reaction.^22^ Since the catalytic effect of the second water molecule observed in CCSD(T)/cc-pVQZ calculation is adequately reproduced by CCSD(T)/cc-pVQZ//DFT/cc-pVTZ calculation, evaluating CCSD(T)/cc-pVQZ energy at the geometry optimized DFT/cc-pVTZ is a good way to analyze Cl· + (H_2_O)_*n*_ → HCl + OH·(H_2_O)_*n*−1_ reactions efficiently. In addition, the relative energy of transition state is 1.2 kcal mol^−1^ lower than products in CCSD(T)/cc-pVQZ results. In other words, the reverse reaction, HCl + OH·(H_2_O) → Cl· + (H_2_O)_2_, has negative activation barrier. Such negative activation barrier of the reverse reaction is also adequately predicted by CCSD(T)/cc-pVQZ//DFT/cc-pVTZ calculations.

**Fig. 3:**
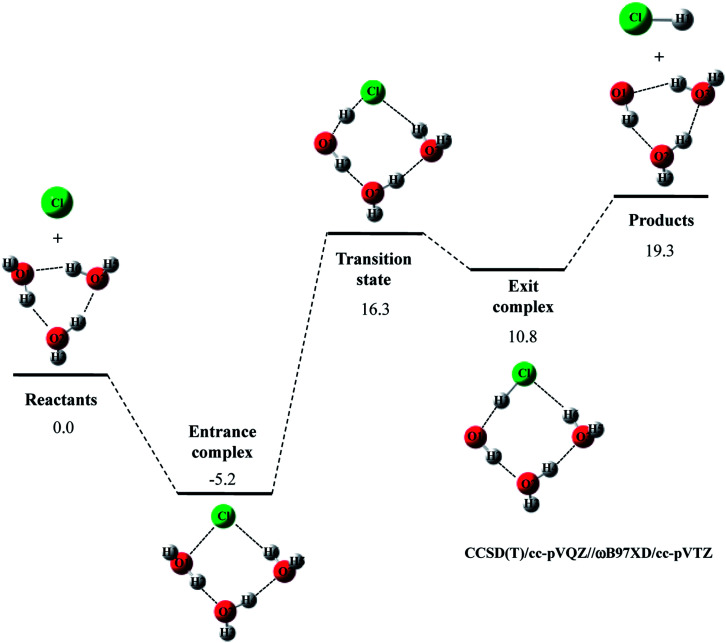
The potential energy profile (kcal mol^−1^) and stationary point structures for Cl· + (H_2_O)_3_ → HCl + OH·(H_2_O)_2_ reaction obtained by CCSD(T)/cc-pVQZ//DFT/cc-pVTZ calculations. Relative energies are calculated as the energy difference between the energy of each stationary point structure and sum of energies of Cl radical and (H_2_O)_3_ (reactants).

The potential energy diagram and optimized structures for Cl· + (H_2_O)_3_ → HCl + OH·(H_2_O)_2_ reaction are shown in Fig. 3, and the optimized geometrical parameters of each stationary point structure are listed in Tables S4–S8 in ESI.† As far as we know, CCSD(T)/cc-pVQZ study on Cl· + (H_2_O)_3_ → HCl + OH·(H_2_O)_2_ reaction has not been reported so far. Since CCSD(T)/cc-pVQZ//ωB97XD/cc-pVTZ calculations gave the reliable potential energy diagrams for the reaction of Cl· with H_2_O and (H_2_O)_2_, as mentioned above, we would like to analyze the reaction of Cl· with (H_2_O)_3_ in the same manner.

In Cl· + (H_2_O)_3_ → HCl + OH·(H_2_O)_2_ reaction, the relative energy of entrance complex and products are slightly higher than those in Cl· + (H_2_O)_2_ → HCl + OH·(H_2_O) reaction, whereas that of transition state is unchanged by existence of the third water molecule. Therefore, the third water molecule hardly act as a catalyst in Cl· + (H_2_O)_3_ → HCl + OH·(H_2_O)_2_ reaction. It should be mentioned that the relative energy of transition state in F· + (H_2_O)_3_ → HF + OH·(H_2_O)_2_ reaction is 1.5 kcal mol^−1^ lower than that in the reaction of F· + (H_2_O)_2_. Thus, unlike Cl· + (H_2_O)_3_ → HCl + OH·(H_2_O)_2_ reaction, the catalytic effect of the third water molecule is observed in F· + (H_2_O)_3_ → HF + OH·(H_2_O)_2_ reaction.^22^

Let us briefly summarize the results of the conventional DFT and CCSD(T)//DFT calculations of Cl· + (H_2_O)_*n*_ → HCl + OH·(H_2_O)_*n*−1_ (*n* = 1–3) reactions. First of all, we have investigated the performance of several density functionals for geometry optimization calculations of the reactions of Cl· with (H_2_O)_*n*_ (*n* = 1 and 2). The B3LYP and M06 calculations tended to overestimate the interatomic O1⋯H1 distance in transition state of Cl· + H_2_O → HCl + OH· reaction, and the MPW1K calculation slightly underestimated covalent O–H bond length in H_2_O monomer. Meanwhile, CAM-B3LYP/cc-pVTZ calculations gave shorter R(O2⋯H2) and R(Cl⋯H3) interatomic distances compared to CCSD(T)/cc-pVQZ ones in entrance complex of Cl· + (H_2_O)_2_ → HCl + OH·(H_2_O) reaction. The CAM-B3LYP calculations, thus, overestimated the interaction between Cl(H_2_O) and the second water molecule. Therefore, our results indicate that the ωB97XD/cc-pVTZ method is the best choice to reproduce the geometries of CCSD(T)/cc-pVQZ stationary point structure in Cl· + (H_2_O)_*n*_ → HCl + OH·(H_2_O)_*n*−1_ (*n* = 1–3) reactions. The potential energy diagrams obtained by CCSD(T)/cc-pVQZ//ωB97XD/cc-pVTZ calculations are indeed close to those obtained by CCSD(T)/cc-pVQZ calculations.^6,7^ Therefore, we have clearly demonstrated that the reliable high-accuracy energies can be obtained by evaluating CCSD(T)/cc-pVQZ energy at the geometries optimized by ωB97XD/cc-pVTZ instead of calculating time-consuming CCSD(T)/cc-pVQZ geometry optimization calculation. Moreover, we have found that the third water molecule hardly catalyze the reaction unlike the second water molecule in Cl· + (H_2_O)_2_ reaction and the third water molecule in F· + (H_2_O)_3_ reaction.^22^

### 4.2. Nuclear quantum effect and H/D isotope effect on Cl· + (H_2_O)_*n*_ → HCl + OH·(H_2_O)_*n*−1_ (*n* = 1–3) reactions

**Fig. 4:**
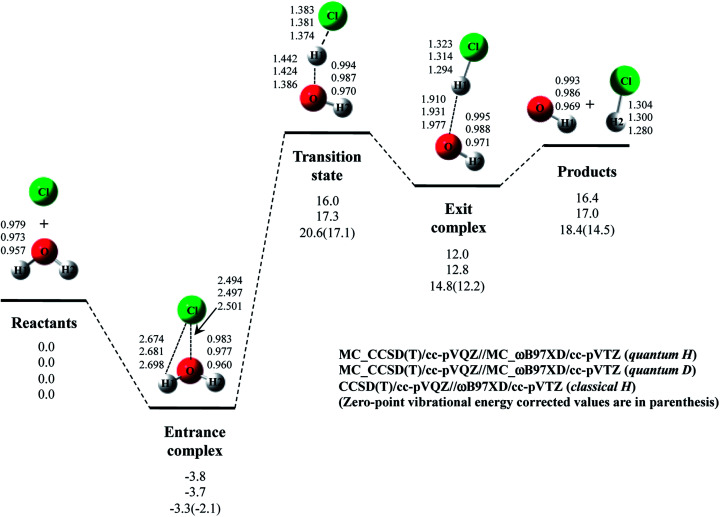
The potential energy profile (kcal mol^−1^) and the optimized geometrical parameters (Å) for Cl· + H_2_O → HCl + OH· and Cl· + D_2_O → DCl + OD· reaction obtained by MC_CCSD(T)/cc-pVQZ//MC_ωB97XD/cc-pVTZ and CCSD(T)/cc-pVQZ//ωB97XD/cc-pVTZ calculations. Relative energies are calculated as the energy difference between the energy of each stationary point structure and sum of energies of Cl radical and H_2_O or D_2_O (reactants).

Next, we performed MC_CCSD(T)/cc-pVQZ//MC_ωB97XD/cc-pVTZ calculations to analyze Cl· + H_2_O → HCl + OH· reaction including NQE of proton. The optimized structures and the potential energy diagram are shown in Fig. 4 (quantum H). We can find that quantum mechanical treatment of proton affects geometrical parameters. For example, the covalent O–H2 and Cl–H1 bond lengths in exit complex of quantum H are 0.995 Å and 1.323 Å, respectively, whereas those are 0.971 Å and 1.294 Å in the conventional ωB97XD result (denoted as classical H). Fig. 4 also shows the results of the reaction with D_2_O (quantum D). In quantum D results, the covalent O–D2 and Cl–D1 bond lengths in exit complex are 0.988 Å and 1.314 Å, respectively, which are shorter than the counterpart of quantum H. These changes of covalent bond lengths are due to the direct inclusion of the anharmonicity of potential energy curve along the covalent bond direction by MC_QM method. On the other hand, the intermolecular O1⋯D1 distance (1.931 Å) in quantum D is slightly longer than the intermolecular O1⋯H1 distance (1.910 Å) in quantum H. We have already revealed that the elongation of intermolecular distances can be understood by the difference between localized nature of protonic and deuteronic wavefunctions.^20^ Since deuteron is twice heavier than proton, deuteronic wavefunction is more localized compared to protonic one. Consequently, deuteronic wavefunction more strongly attracts surrounding electrons, and deuterium becomes less positive and acts as weaker hydrogen nucleus-donor than hydrogen. In fact, the optimized orbital exponent *α* values for H1 and H2 of exit complex are 20.6 and 22.8, and those for D1 and D2 are 30.9 and 34.1, respectively. (see, Fig. S3 in ESI†). Since larger *α* value represents more localized nuclear wavefunction, deuteronic wavefunctions are indeed more localized than protonic ones in our MC_QM calculations. In addition, the NBO charges on proton and deuteron are 0.154 and 0.150 in quantum H and quantum D, respectively. Deuteron is indeed less positive than proton.

Next, we would like to focus on NQE on the potential energy profiles of Cl· + H_2_O → HCl + OH· reaction (Fig. 4). The relative energy of quantum H is always lower than that of quantum D and classical H. In other words, NQE of proton lowered the relative energies of all stationary point structures. In particular, larger stabilization by NQE is observed in transition state and exit complex due to the hydrogen-bonded interaction. Larger stabilization appears in quantum H rather than in quantum D, because NQE of heavier nucleus (deuteron) is smaller than that of lighter one (proton). In particular, the relative energy of transition state of quantum H is 0.4 kcal mol^−1^ lower than products. The reverse reaction HCl + OH· → Cl· + H_2_O, thus, has a negative activation barrier when considering NQE of proton, even only one water molecule participate in the reaction. We should note here that harmonic zero-point vibration energy correction (also shown in Fig. 4) lower not only transition state but also products. The harmonic ZPVE correction, thus, did not remove the activation barrier of the reverse reaction.

To analyze the stabilization by NQE in detail, we additionally performed the MC_CCSD(T)/cc-pVQZ energy calculation at the geometry optimized by ωB97XD/cc-pVTZ. (MC_CCSD(T)/cc-pVQZ//ωB97XD/cc-pVTZ) for Cl· + H_2_O → HCl + OH· reaction. In such calculation, the geometrical relaxation induced by NQE is not taken into account, whereas the nuclear quantum fluctuation of proton is represented in MC_CCSD(T) energy calculation. The calculated energy diagram is shown in Fig. S4 in ESI.† Although the relative energies obtained in MC_CCSD(T)/cc-pVQZ//ωB97XD/cc-pVTZ calculations are slightly (0.5–0.7 kcal mol^−1^) higher than those obtained in MC_CCSD(T)/cc-pVQZ//MC_ωB97XD/cc-pVTZ calculations, the potential energy profiles obtained in MC_CCSD(T)/cc-pVQZ//ωB97XD/cc-pVTZ and MC_CCSD(T)/cc-pVQZ//MC_ωB97XD/cc-pVTZ calculations are almost parallel to each other. In addition, the activation barrier for the reverse reaction is also negative in MC_CCSD(T)/cc-pVQZ//ωB97XD/cc-pVTZ result. These results show that the direct quantum mechanical treatment of light nuclei by appropriate method, such as our MC_QM, is indispensable to analyze potential energy profiles for Cl· + H_2_O → HCl + OH· reaction adequately.

**Fig. 5:**
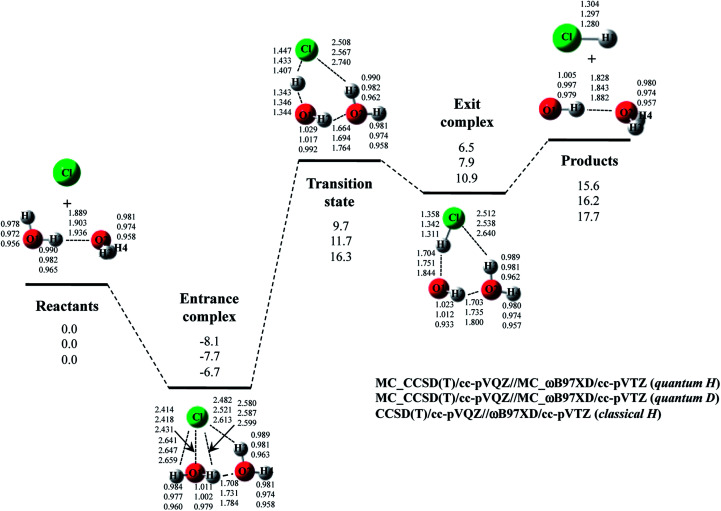
The potential energy profile (kcal mol^−1^) and the optimized geometrical parameters (Å) for Cl· + (H_2_O)_2_ → HCl + OH·(H_2_O) and Cl· + (D_2_O)_2_ → DCl + OD·(D_2_O) reaction obtained by MC_CCSD(T)/cc-pVQZ//MC_ωB97XD//cc-pVTZ and CCSD(T)/cc-pVQZ//ωB97XD/cc-pVTZ calculations. Relative energies are calculated as the energy difference between the energy of each stationary point structure and sum of energies of Cl radical and (H_2_O)_2_ or (D_2_O)_2_ (reactants).

The optimized structure and potential energy profiles for Cl· + (H_2_O)_2_ → HCl + OH·(H_2_O) and Cl· + (D_2_O)_2_ → DCl + OD·(D_2_O) reactions are shown in Fig. 5. The covalent bond lengths and the intermolecular distances are the shortest and the longest in quantum H, respectively, as well as the reaction with one H_2_O molecule. Meanwhile, NQE of proton and deuteron seemingly did not affect the intermolecular O1⋯H1 and O1^…^D1 distances in transition state, since the intermolecular O1⋯H1(D1) distances in transition state in quantum H, quantum D, and classical H are very similar to each other. Since H1 atom is located in the almost midpoint between O1 and Cl, H1 atom is loosely bounded to both O1 and Cl in transition state. Hence, the O1⋯H1 distances do not show the typical H/D geometrical isotope effect in transition state. We would like to advocate that NQE of proton and deuteron also provide important effect on transition state because other parameters, such as O1–H2, O2–H3, O2–H4, O2⋯H2, and Cl⋯H3, are affected by NQE, and the spatial distributions of protonic and deuteronic wavefunctions are different from each other (see, optimized exponent *α*_opt_ value in Cl· + (H_2_O)_2_ → HCl + OH·(H_2_O) and Cl· + (D_2_O)_2_ → DCl + OD·(D_2_O) reactions in Fig. S5 in ESI†).

Next, we would like to focus on the potential energy profile of Cl· + (H_2_O)_2_ → HCl + OH·(H_2_O) and Cl· + (D_2_O)_2_ → DCl + OD·(D_2_O) reactions. As in the case of classical H, the second water molecule lowered the relative energies of all stationary points compared to the reaction with one H_2_O (or D_2_O) molecule. NQEs of proton and deuteron stabilize all the stationary point structures in quantum H and quantum D. The relatively large stabilization effects appear in transition state and exit complex due to the number of hydrogen-bonded interactions, as well as the reactions with one water molecule. Consequently, NQE of proton and deuteron lower the activation barrier of Cl· + (H_2_O)_2_ → HCl + OH·(H_2_O) and Cl· + (D_2_O)_2_ → DCl + OD·(D_2_O) reactions.

**Fig. 6:**
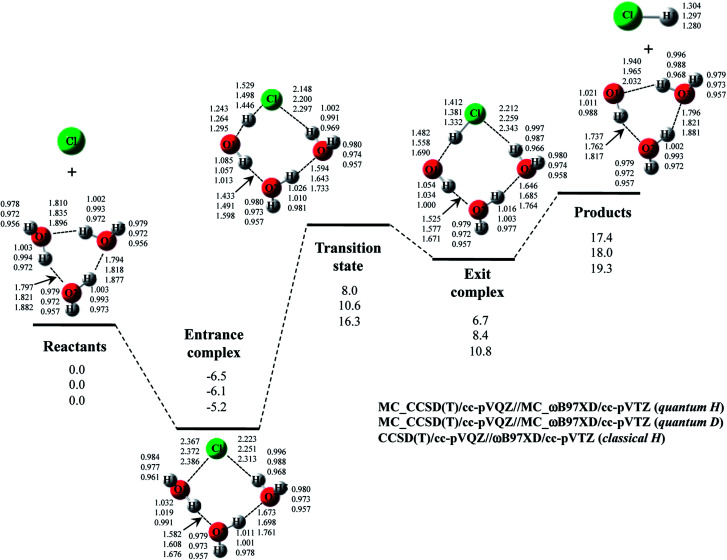
The potential energy profile (kcal mol^−1^) and the optimized geometrical parameters (Å) for Cl· + (H_2_O)_3_ → HCl + OH·(H_2_O)_2_ and Cl· + (D_2_O)_3_ → DCl + OD·(D_2_O)_2_ reaction obtained by MC_CCSD(T)/cc-pVQZ//MC_ωB97XD/cc-pVTZ and CCSD(T)/cc-pVQZ//ωB97XD/cc-pVTZ calculations. Relative energies are calculated as the energy difference between the energy of each stationary point structure and sum of energies of Cl radical and (H_2_O)_3_ or (D_2_O)_3_ (reactants).

The optimized structures and potential energy profiles for Cl· + (H_2_O)_3_ → HCl + OH·(H_2_O)_2_ and Cl· + (D_2_O)_3_ → DCl + OD·(D_2_O)_2_ are shown in Fig. 6 (see, Fig. S6 in ESI† for the optimized exponent *α*_opt_ values). NQE also impacts the geometrical parameters of the stationary point structures, like the reactions with one or two H_2_O (or D_2_O) molecule(s). The covalent bond length and interatomic distances were elongated and shortened by including NQE, respectively.

Due to the difference of the number of hydrogen-bonded interactions in the structure, the greater stabilizations are again found in transition state and exit complex rather than in entrance complex. As mentioned in Sec. 4-1, the relative energies of transition state in Cl· + (H_2_O)_2_ and Cl· + (H_2_O)_3_ reactions of classical H are the same (16.3 kcal mol^−1^). The catalytic effect of the third water molecule is, thus, not observed in classical H calculation. However, the relative energies of transition state in quantum H and quantum D are 1.7 kcal mol^−1^ and 1.1 kcal mol^−1^ lower than those in the reactions with two water molecules, respectively. Therefore, we can find that the clear catalytic effect of the third water molecule appears when NQEs of proton and deuteron are adequately taken into account. The potential energy diagrams of Cl· + (H_2_O)_2_ and Cl· + (H_2_O)_3_ reactions corrected for ZPVE are shown in Fig. S7 and S8 in ESI† for comparison. The ZPVE-corrected relative energy of transition state in Cl· + (H_2_O)_3_ reaction is lower than that in Cl· + (H_2_O)_2_ one. However, ZPVE correction destabilizes the relative energy of entrance complex. This tendency is opposite to the MC_QM results. Thus, we claim to that the geometrical relaxation effect is also important to adequately analyze the catalytic effect of water molecule(s) on the reaction.

Let us summarize NQE on the reactions of Cl· + (H_2_O)_*n*_ (*n* = 1–3) briefly. We have analyzed the influence of NQE of proton and deuteron on the stationary point structures and potential energy profiles of Cl· + (H_2_O)_*n*_ → HCl + OH·(H_2_O)_*n*−1_ and Cl· + (D_2_O)_*n*_ → DCl + OD·(D_2_O)_*n*−1_ (*n* = 1–3) reactions using MC_QM method. We have clearly demonstrated that NQEs of hydrogen nuclei lower the relative energies of all stationary point structures in Cl· + (H_2_O)_*n*_ → HCl + OH·(H_2_O)_*n*−1_ (*n* = 1–3) and its deuterated reactions. Furthermore, the activation barriers for these reactions (relative energy of transition state) are also lowered by NQE. In particular, the relative energy of transition state of Cl· + H_2_O → HCl + OH· reaction lies below the separated products molecules in quantum H. Thus, the reverse reaction of Cl· + H_2_O → HCl + OH· has negative activation barrier when considering NQE of proton. In addition, our calculation suggests that the catalytic effect of the third water molecule only appears when NQEs of proton and deuteron are adequately taken into account.

## 5. Conclusion

In this study, we analyzed Cl· + (H_2_O)_*n*_ → HCl + OH·(H_2_O)_*n*−1_ and Cl· + (D_2_O)_*n*_ → DCl + OD·(D_2_O)_*n*−1_ (*n* = 1–3) reactions using MC_QM method, which can directly reflect NQEs of light nuclei on electronic states. First, we investigated the performance of five major density functionals to efficiently reproduce the CCSD(T)/cc-pVQZ structure. As a result, ωB97XD/cc-pVTZ calculation gave the best structure among these five functionals. Next, we investigated the NQE of proton and deuteron on the optimized structures and energy diagram of the reactions using MC_QM method, and find that NQE of hydrogen nuclei lower the relative energies of the stationary point structures. In particular, NQE significantly lowers the activation barrier of Cl· + (H_2_O)_*n*_ → HCl + OH·(H_2_O)_*n*−1_ (*n* = 1–3) reactions, and consequently the relative energy of transition state in Cl· + H_2_O → HCl + OH· is lower than that of separated products when NQE of proton is directly considered. Furthermore, although the conventional DFT calculations gave the same activation energies for the reactions with (H_2_O)_2_ and (H_2_O)_3_, the activation energy in the reaction with (H_2_O)_3_ is lower than that with (H_2_O)_2_ in the results obtained with MC_QM method. In other words, the catalytic effect of the third water molecule is observed in only MC_QM calculations. Therefore, we clearly demonstrate the importance of direct inclusion of protonic and deuteronic quantum natures to analyze Cl· + (H_2_O)_*n*_ → HCl + OH·(H_2_O)_*n*−1_ (*n* = 1–3) reactions, as well as the reactions of F· + (H_2_O)_*n*_.^22^

## Conflicts of interest

There are no conflicts of interest to declare.

## Supplementary Material

RA-008-C8RA02679C-s001
