# Supplementary material for: Nuclear quantum effect and H/D isotope effect on Cl· + (H2O)n → HCl + OH·(H2O)n−1 (n = 1–3) reactions
Source: RSC Adv. 2018 May 10;8(31):17191–201. doi: 10.1039/c8ra02679c (PMC9080416; doi:10.1039/c8ra02679c)
Supplement: RA-008-C8RA02679C-s001 [file RA-008-C8RA02679C-s001.pdf]

Electronic Supplementary Material (ESI) for RSC Advances.
This journal is © The Royal Society of Chemistry 2018

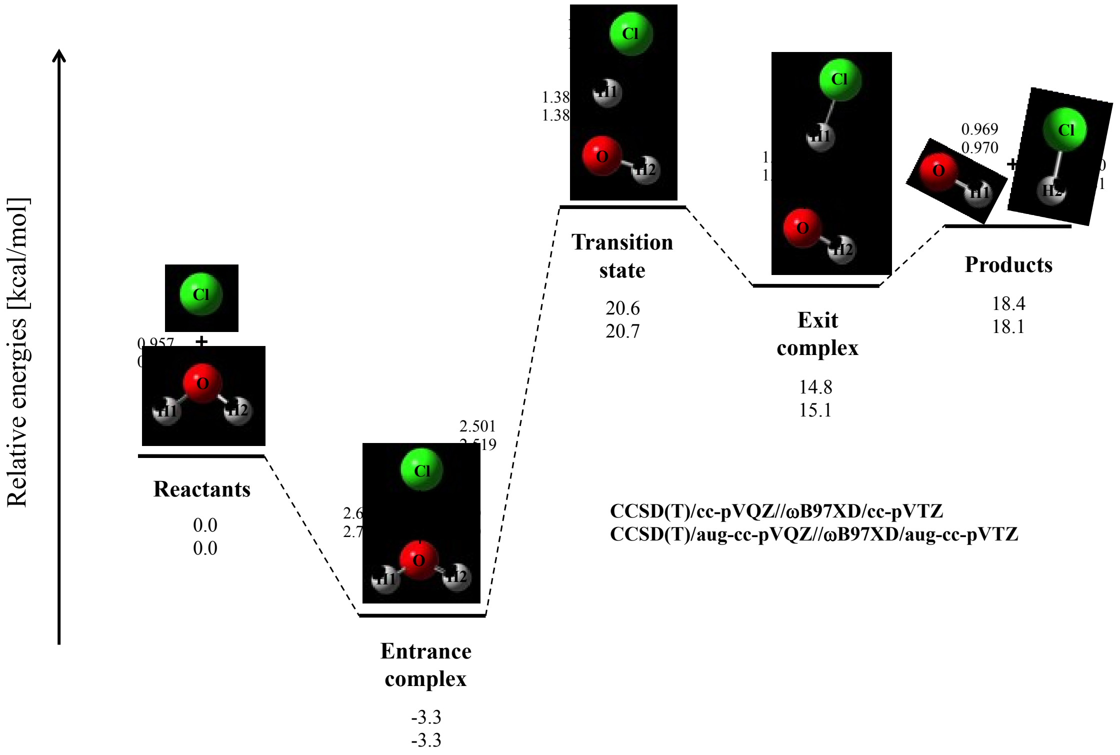

FIGURE S1. The energy profile [kcal/mol] and geometrical parameters for Cl· + H_2O → HCl + OH· reaction obtained by CCSD(T)/cc-pVQZ//ωB97XD/cc-pVTZ and CCSD(T)/aug-cc-pVQZ//ωB97XD/aug-cc-pVTZ calculations.

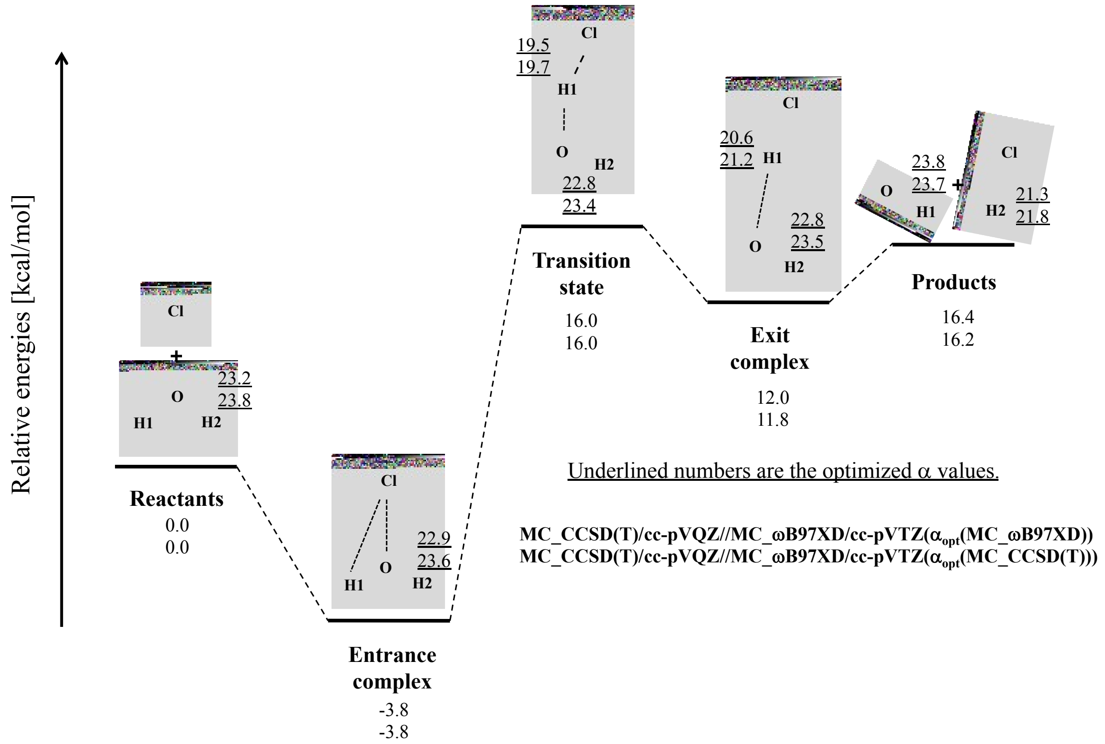

FIGURE S2. The potential energy profile [kcal/mol] for Cl· + H_2O → HCl + OH· reaction by MC_CCSD(T)/cc-pVQZ//MC_ωB97XD/cc-pVTZ calculations with the optimized α_{opt} values by MC_ωB97XD/MC_cc-pVQZ//MC_ωB97XD/cc-pVTZ(α_{opt}(MC_ωB97XD)) and MC_CCSD(T)/cc-pVQZ//MC_ωB97XD/cc-pVTZ(α_{opt}(MC_CCSD(T))) calculations.

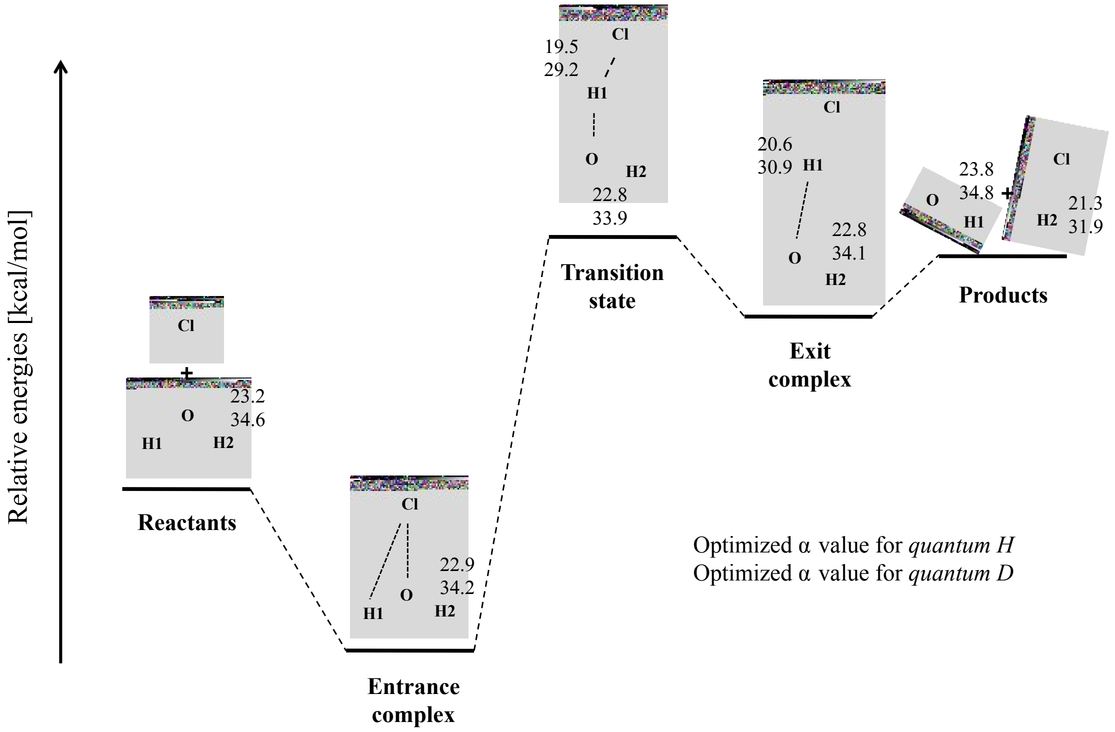

FIGURE S3. The exponent α_{opt} value of nuclear basis function obtained by MC_ωB97XD/cc-pVQZ//MC_ωB97XD/cc-pVTZ calculation for $Cl\cdot + H_2O \rightarrow HCl + OH\cdot$ and $Cl\cdot + D_2O \rightarrow DCl + OD\cdot$ reaction.

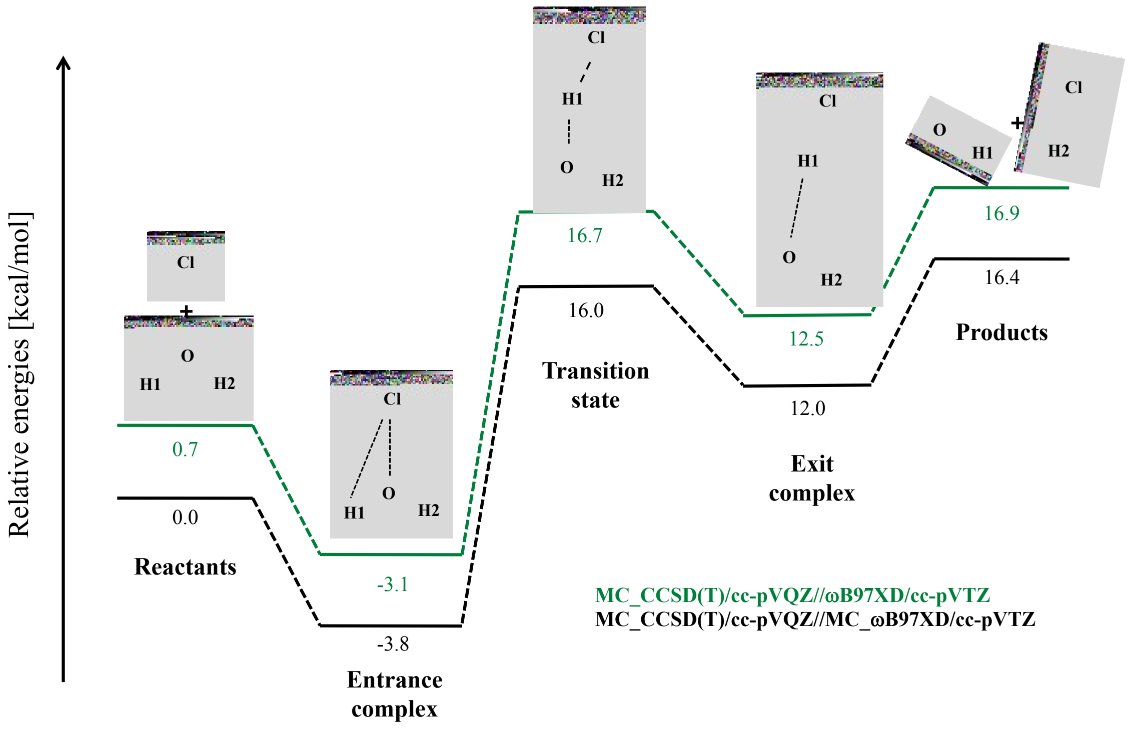

FIGURE S4. The potential energy profile [kcal/mol] for Cl· + H_2O → HCl + OH· reaction obtained by MC_CCSD(T)/cc-pVQZ//ωB97XD/cc-pVTZ and MC_CCSD(T)/cc-pVQZ//MC_ωB97XD/cc-pVTZ methods.

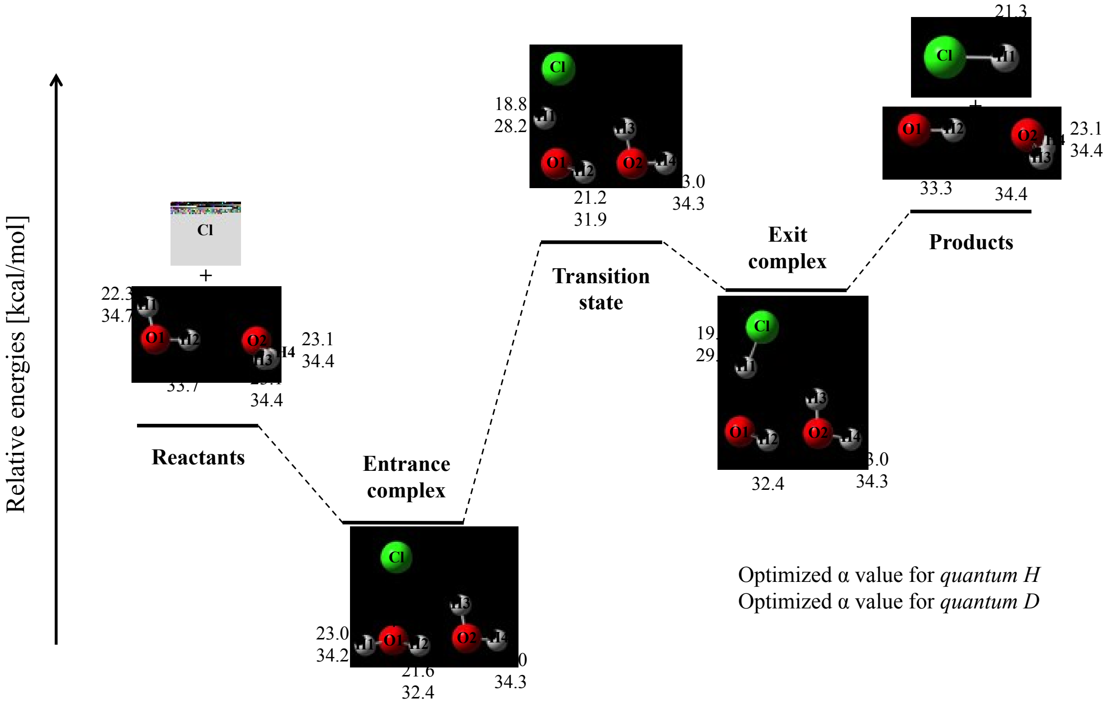

FIGURE S5. The exponent α_{opt} value of nuclear basis function obtained by MC_ωB97XD.cc-pVQZ//MC_ωB97XD/cc-pVTZ calculation for Cl· + $(H_2O)_2$ → HCl + OH·(H_2O) and Cl· + $(D_2O)_2$ → DCl + OD·(D_2O) reaction.

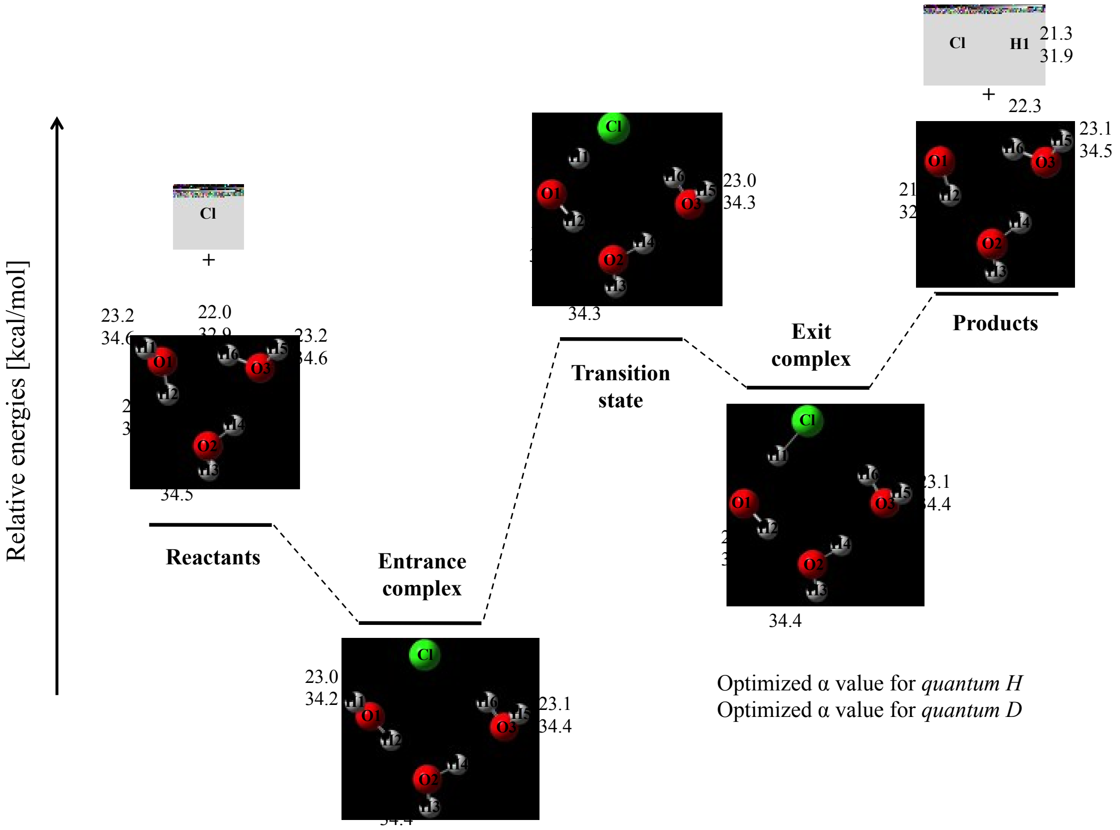

FIGURE S6. The exponent α_{opt} value of nuclear basis function obtained by MC_ωB97XD/cc-pVQZ//MC_ωB97XD/cc-pVTZ calculation for $Cl\cdot + (H_2O)_3 \rightarrow HCl + OH\cdot(H_2O)_2$ and $Cl\cdot + (D_2O)_3 \rightarrow DCl + OD\cdot(D_2O)_2$ reaction.

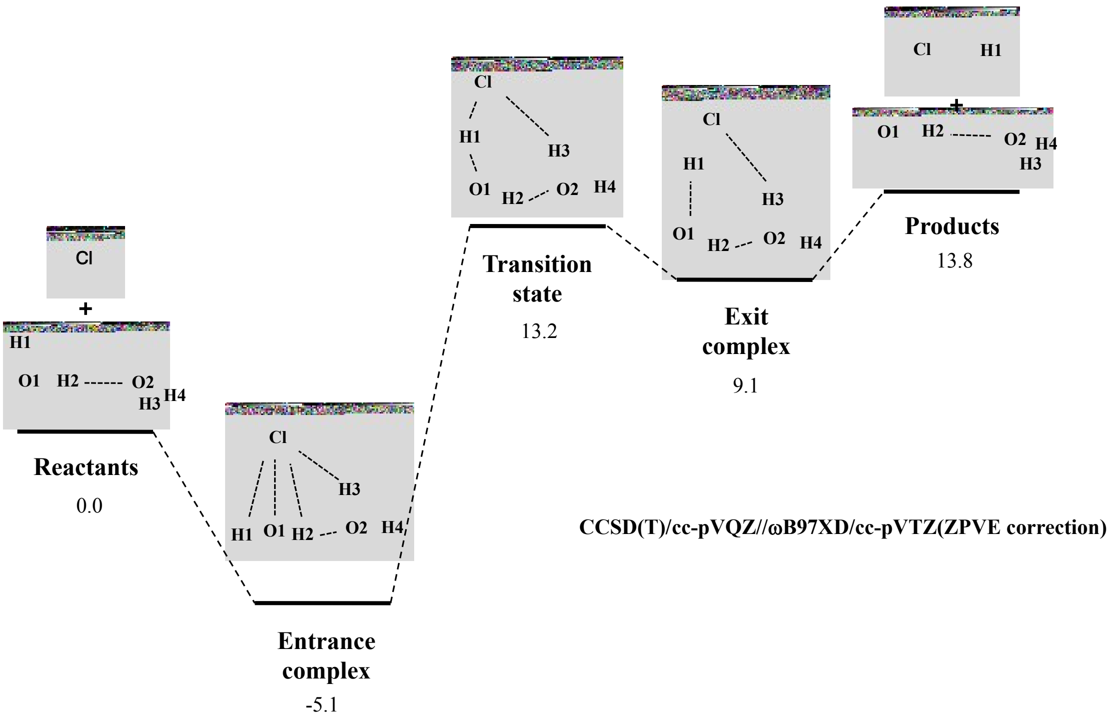

FIGURE S7. The potential energy profile (kcal/mol) and the optimized geometrical parameters (Å) for Cl· + $(H_2O)_2$ → HCl + OH· (H_2O) reaction obtained by CCSD(T)/cc-pVQZ//ωB97XD/cc-pVTZ calculations. Relative energies are calculated as the energy difference between the energy of each stationary point structure and sum of energies of Cl radical and $(H_2O)_2$ (**Reactants**).

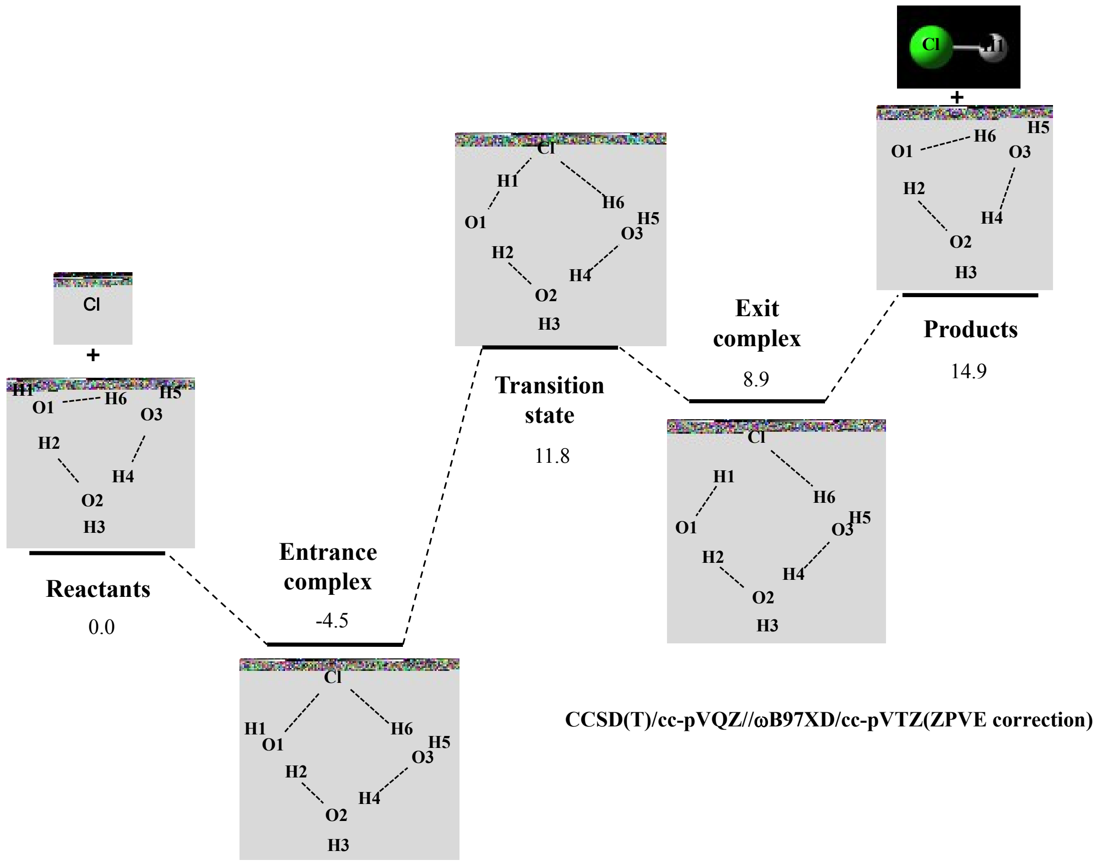

FIGURE S8. The potential energy profile (kcal/mol) and stationary point structures for Cl· + $(H_2O)_3$ → HCl + OH· $(H_2O)_2$ reaction obtained by CCSD(T)/cc-pVQZ//ωB97XD/cc-pVTZ calculations. Relative energies are calculated as the energy difference between the energy of each stationary point structure and sum of energies of Cl radical and $(H_2O)_3$ (**Reactants**).

TABLE S1. Optimized geometrical parameter (Å) of **Reactants** for Cl· + $(H_2O)_2$ → HCl + OH·(H_2O) reaction obtained by DFT/cc-pVTZ methods.

Method	R(O1-H1)	R(O1-H2)	R(O2-H3)	R(O2···H2)
**B3LYP**	0.960	0.968	0.962	1.948
**CAM-B3LYP**	0.959	0.967	0.961	1.906
**M06**	0.957	0.966	0.959	1.936
**ωB97XD**	0.956	0.965	0.958	1.936
**MPW1K**	0.950	0.958	0.951	1.933
**CCSD(T)/cc-pVQZ**[a]	0.957	0.964	0.959	1.953

[a]Ref. 7

TABLE S2. Optimized geometrical parameter (Å) of **Exit complex** for Cl· + $(H_2O)_2$ → HCl + OH·(H_2O) reaction obtained by DFT/cc-pVTZ methods.

Method	R(O1-H2)	R(O2-H3)	R(O2-H4)	R(O1···H1)	R(O2···H2)	R(Cl···H1)	R(Cl···H3)
**B3LYP**	0.994	0.967	0.962	1.818	1.788	1.318	2.649
**CAM-B3LYP**	0.993	0.966	0.960	1.810	1.761	1.313	2.582
**M06**	0.989	0.965	0.958	1.877	1.809	1.311	2.514
**ωB97XD**	0.988	0.962	0.957	1.844	1.800	1.311	2.640
**MPW1K**	0.981	0.955	0.951	1.813	1.779	1.304	2.661
**CCSD(T)/ cc-pVQZ**[a]	0.983	0.962	0.968	1.916	1.835	1.298	2.616

[a]Ref. 7

TABLE S3. Optimized geometrical parameter (Å) of **Products** for Cl· + $(H_2O)_2$ → HCl + OH·(H_2O) reaction obtained by DFT/cc-pVTZ methods.

	**OH·(H_2O)**			**HCl**
**Method**	R(O1-H2)	R(O2-H3)	R(O2···H2)	R(Cl-H2)
**B3LYP**	0.983	0.962	1.881	1.283
**CAM-B3LYP**	0.982	0.960	1.848	1.280
**M06**	0.980	0.958	1.886	1.283
**ωB97XD**	0.979	0.957	1.882	1.280
**MPW1K**	0.971	0.951	1.871	1.273
**CCSD(T)/cc-pVQZ[a]**	0.976	0.958	1.903	1.277

[a]Ref. 7

TABLE S4. Optimized geometrical parameters (Å) of **Reactants** for Cl· + $(H_2O)_3$ → HCl + OH·$(H_2O)_2$ reaction obtained by DFT/cc-pVTZ methods.

Method	R(O1-H1)	R(O1-H2)	R(O2-H3)	R(O2-H4)	R(O3-H5)	R(O3-H6)	R(O1···H6)	R(O2···H2)	R(O3···H4)
**B3LYP**	0.961	0.977	0.961	0.977	0.961	0.977	1.894	1.877	1.876
**CAM-B3LYP**	0.959	0.977	0.959	0.977	0.959	0.976	1.856	1.841	1.837
**M06**	0.957	0.974	0.957	0.974	0.957	0.974	1.891	1.882	1.876
**ωB97XD**	0.956	0.972	0.957	0.973	0.956	0.972	1.896	1.882	1.877
**MPW1K**	0.950	0.966	0.950	0.966	0.950	0.965	1.882	1.862	1.859

TABLE S5. Optimized geometrical parameters (Å) of **Entrance complex** for Cl· + $(H_2O)_3$ → HCl + OH·$(H_2O)_2$ reaction obtained by DFT/cc-pVTZ methods.

Method	R(O1-H1)	R(O1-H2)	R(O2-H3)	R(O2-H4)	R(O3-H5)	R(O3-H6)	R(O1···Cl)	R(O2···H2)	R(O3···H4)	R(Cl···H6)
**B3LYP**	0.965	1.000	0.961	0.984	0.962	0.973	2.419	1.660	1.752	2.311
**CAM-B3LYP**	0.963	0.997	0.960	0.982	0.960	0.971	2.371	1.649	1.730	2.295
**M06**	0.962	0.990	0.958	0.978	0.958	0.969	2.433	1.706	1.776	2.331
**ωB97XD**	0.960	0.991	0.957	0.978	0.957	0.968	2.386	1.676	1.761	2.313
**MPW1K**	0.953	0.984	0.951	0.971	0.951	0.961	2.356	1.664	1.749	2.320

TABLE S6. Optimized geometrical parameters (Å) of **Transition state** for Cl· + $(H_2O)_3$ → HCl + OH·$(H_2O)_2$ reaction obtained by DFT/cc-pVTZ methods.

Method	R(O1-H2)	R(O2-H3)	R(O2-H4)	R(O3-H5)	R(O3-H6)	R(O1···H1)	R(O2···H2)	R(O3···H4)	R(Cl···H1)	R(Cl···H6)
**B3LYP**	1.017	0.961	0.985	0.962	0.973	1.379	1.611	1.739	1.418	2.324
**CAM-B3LYP**	1.022	0.960	0.986	0.960	0.972	1.289	1.563	1.700	1.452	2.273
**M06**	1.001	0.958	0.980	0.958	0.970	1.374	1.631	1.754	1.420	2.289
**ωB97XD**	1.013	0.957	0.981	0.957	0.969	1.295	1.598	1.733	1.446	2.297
**MPW1K**	1.018	0.951	0.978	0.951	0.964	1.197	1.532	1.694	1.503	2.255

TABLE S7. Optimized geometrical parameters (Å) of **Exit complex** for Cl· + $(H_2O)_3$ → HCl + OH·$(H_2O)_2$ reaction obtained by DFT/cc-pVTZ methods.

Method	R(O1-H2)	R(O2-H3)	R(O2-H4)	R(O3-H5)	R(O3-H6)	R(Cl-H1)	R(O1···H1)	R(O2···H2)	R(O3···H4)	R(Cl···H6)
**B3LYP**	1.008	0.961	0.983	0.962	0.971	1.341	1.668	1.659	1.760	2.361
**CAM-B3LYP**	1.007	0.960	0.982	0.960	0.970	1.335	1.662	1.636	1.732	2.326
**M06**	0.998	0.957	0.978	0.958	0.968	1.325	1.752	1.701	1.783	2.343
**ωB97XD**	1.000	0.957	0.977	0.958	0.966	1.332	1.690	1.671	1.764	2.343
**MPW1K**	0.994	0.950	0.971	0.951	0.960	1.326	1.661	1.652	1.751	2.350

TABLE S8. Optimized geometrical parameters (Å) of **Products** for Cl· + $(H_2O)_3$ → HCl + OH·$(H_2O)_2$ reaction obtained by DFT/cc-pVTZ methods

	**OH·$(H_2O)_2$**								**HCl**
**Method**	R(O1-H2)	R(O2-H3)	R(O2-H4)	R(O3-H5)	R(O3-H6)	R(O1···H6)	R(O2···H2)	R(O3···H4)	R(Cl-H1)
**B3LYP**	0.994	0.961	0.977	0.961	0.973	2.018	1.808	1.878	1.283
**CAM-B3LYP**	0.994	0.959	0.976	0.959	0.972	1.977	1.775	1.838	1.280
**M06**	0.989	0.957	0.973	0.957	0.970	2.006	1.822	1.877	1.283
**ωB97XD**	0.988	0.957	0.972	0.957	0.968	2.032	1.817	1.881	1.280
**MPW1K**	0.980	0.950	0.965	0.950	0.961	2.018	1.799	1.865	1.273
